# Challenges in the diagnosis and treatment of genetic cholestasis in adults

**DOI:** 10.1016/j.jhepr.2025.101625

**Published:** 2025-10-09

**Authors:** Richard J. Thompson, Silvia Vilarinho, Rosa Miquel, Verena Keitel

**Affiliations:** 1 Roger Williams Institute of Liver Studies, King's College London, London, UK; 2 Department of Internal Medicine, Section of Digestive Diseases, Yale School of Medicine, New Haven, Connecticut, USA; 3 Departments of Genetics and Pathology, Yale School of Medicine, New Haven, Connecticut, USA; 4 Institute of Liver Studies, Liver Histopathology Laboratory, King's College Hospital, London, UK; 5 Department of Gastroenterology, Hepatology and Infectious Diseases, University Hospital Magdeburg Otto von Guericke University, Magdeburg, Germany

**Keywords:** Primary cholestasis, cholangiopathy, gamma glutamyl transferase, bile acids, bile salt export pump

## Abstract

Disorders of bile formation and bile flow along the intra- and extrahepatic bile ducts are summarised under the term cholestasis. Clinically, conditions resulting in retention of biliary constituents such as bile acids within hepatocytes (termed primary cholestasis) need to be distinguished from diseases characterised by bile duct injury (termed cholangiopathies). Some cholangiopathies may also cause retention of biliary constituents within hepatocytes, resulting in secondary cholestasis. Genetic variants in a multitude of genes can contribute to the development of both primary cholestasis and cholangiopathies. Assessing the contribution of identified genetic variants to the clinical presentation in adults is complicated by factors such as environmental exposure, comorbidities, and medication intake. The diagnostic workup in adults with cholestasis should first consider common causes of primary cholestasis and cholangiopathies. If the aetiology remains unclear, liver histology and/or genetic testing should be pursued. Until recently, treatment for these conditions was largely supportive. However pharmacological interruption of the enterohepatic circulation of bile acids now offers the possibility of more specific intervention. Moreover, for those conditions in which the bile duct epithelium is the main site of injury, ursodeoxycholic acid remains essential. Multidisciplinary case discussions can help facilitate diagnosis and guide management.

## Graphical abstract

**Figure:**
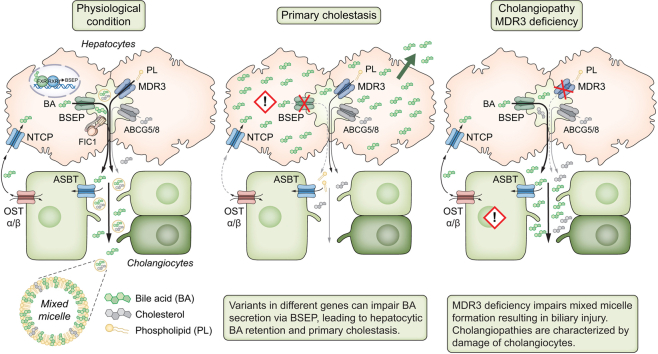

**Keypoints**

• Genetic defects are increasingly recognised as causative or relevant contributing factors in adult primary cholestasis and cholangiopathies.
• Primary cholestasis is defined by retention of biliary constituents such as bile acids within hepatocytes, leading to elevation of serum bile acids despite normal/low gamma-glutamyltransferase levels.
• Cholangiopathies are characterised by injury of the bile ducts and usually associated with an elevation of serum gamma-glutamyltransferase levels.
• A stepwise diagnostic approach with initial assessment of common causes of cholestasis, followed by liver biopsy and genetic testing if aetiology remains unclear, is suggested.
• Multidisciplinary case discussions help to facilitate diagnosis and therapeutic management.
• Treatment of the underlying pathology is not yet possible for genetic cholestasis; however, therapeutic management should alleviate the consequences of cholestasis and include symptom control.

## Introduction

Cholestasis refers to impaired bile formation or disrupted bile flow through the biliary tree into the small intestine. Bile formation involves the synthesis of its constituents, their transport across the canalicular membrane, and movement through the biliary tree.^1^ Mutations in genes vital for the transport of biliary constituents and/or the stability/composition of the apical membranes of hepatocytes and cholangiocytes have been identified in patients with progressive familial intrahepatic cholestasis (PFIC), a severe group of disorders that present in early infancy with jaundice, failure to thrive, pruritus and progressive cholestatic liver disease.^2^ Genetic cholestatic liver diseases have long been considered primarily paediatric disorders, as they were initially described in children. However, with advances in next-generation sequencing technologies and the adoption of whole-exome sequencing (WES) in clinical hepatology,^3^ it has become evident that these diseases can also present in adulthood. Several studies have demonstrated that a substantial proportion of adults with unexplained cholestasis, despite comprehensive evaluation, harbour genetic defects that explain their condition.[4], [5], [6], [7], [8] The recognition of monogenic defects as the cause of cholestatic liver disease in adults has only recently gained attention. One of the challenges in adult genetic cholestasis is the broad spectrum of clinical presentations ranging from elevated liver enzymes, pruritus, infrequent symptoms precipitated by factors such as infection, hormonal status or drug intake to severe early-onset or late-onset progressive disease.^2^ With broader application of genetic testing in liver diseases of unclear origin, genetic contributions will be increasingly identified.^4^^,^^5^^,^^7^ However, determining the extent to which identified genetic variants contribute to the clinical presentation in adults is often challenging, as it is confounded by environmental factors, comorbidities, and medication use.

In this review, we will examine the genetic contribution of PFIC-associated genes to adult primary cholestasis and cholangiopathies, and discuss current therapeutic approaches. Bile acid synthesis defects and cholestasis syndromes including Alagille syndrome, arthrogryposis–renal dysfunction–cholestasis, neonatal sclerosing cholangitis and osteo-oto-hepato-enteric syndrome are beyond the scope of this review and are summarised elsewhere.^9^^,^^10^

## Genes associated with hereditary cholestasis in adults

**Fig. 1:**
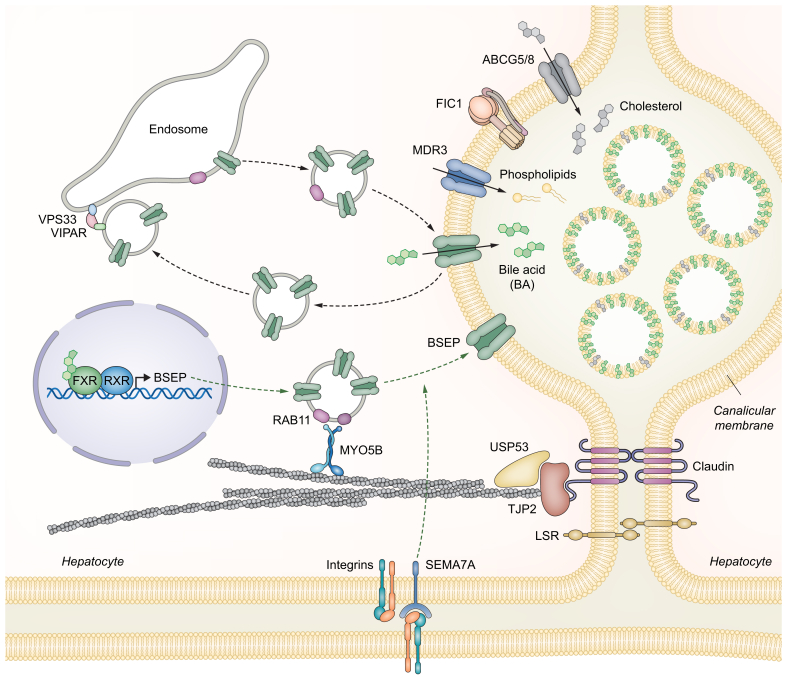
Proteins expressed within the hepatocytes and associated with primary cholestasis (modified from^136^). PFIC-associated genes encode hepatobiliary ATPases and transport proteins, such as the P-type ATPase FIC1 (*ATP8B1*), the bile salt export pump BSEP (*ABCB11*) and the phospholipid floppase MDR3 (*ABCB4*) as well as the nuclear receptor farnesoid X receptor (FXR). Moreover, components of the cell adhesion complex – such as tight junction protein 2 (TJP2/ZO-2), USP53, and LSR – as well as proteins involved in the intracellular trafficking of canalicular transporters, including myosin 5B (MYO5B), VSP33B, VIPAR, and semaphorin 7A (SEMA7A), may also contribute to genetic cholestasis. Bile acids transported by BSEP and phospholipids transported by MDR3 into bile form mixed micelles with cholesterol, which is excreted by ABCG5/G8. For references see text.

**Fig. 2:**
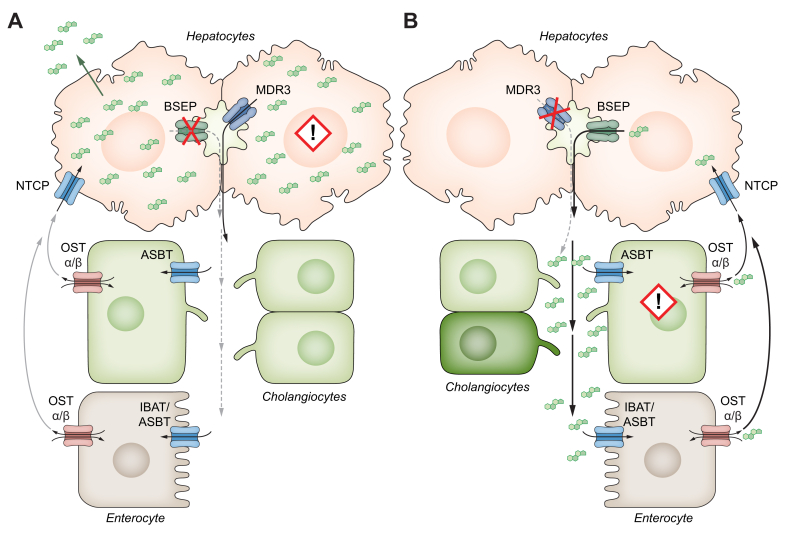
Mechanisms of cholestasis taken from.^137^ (A) Schematic representation of primary cholestasis exemplified by BSEP deficiency. Mutations in a variety of genes reduce the amount of functionally active BSEP within in the canalicular membrane resulting in intrahepatic retention of BAs and hepatocytic damage. (B) Schematic representations of ABCB4-disease. Reduction of phosphatidylcholine secretion into bile reduces the capacity to form mixed micelles leading to a relative increase in toxic bile acids. The latter triggers progressive cholangiocyte damage.

The number of gene defects associated with severe primary cholestasis and cholangiopathies continues to increase due to broader availability of exome and genome sequencing technologies. Ibrahim *et al.*^9^ provide a comprehensive overview of the genes underlying a variety of monogenic cholestasis disorders. Mechanistically, genetic defects resulting in retention of biliary constituents such as bile acids (BAs) within hepatocytes (termed primary cholestasis, Fig. 1, Fig. 2A) will be distinguished from genetic defects characterised by bile duct injury (termed cholangiopathies, Fig. 2B).

### Genes associated with low-GGT primary cholestasis

Primary cholestasis is associated with genes encoding proteins affecting hepatobiliary transport protein expression (*e.g*. *ABCB11, NR1H4*), integrity of the canalicular membrane and tight junctions (*e.g. ATP8B1, TJP2, USP53, LSR*), and intracellular trafficking of hepatobiliary transport proteins (*e.g. MYO5B, SEMA7A*) (Table 1).

**Table 1:** Genes associated with primary cholestasis and typically characterised by normal GGT levels (modified from^9^).

Gene	Protein	Clinical manifestation	Extrahepatic manifestations
*ATP8B1*	FIC1	PFIC, (episodic) cholestasis, ICP, elevated LFT, pruritus	Intestinal symptoms (diarrhoea), hearing loss, pancreatitis
*ABCB11*	BSEP	PFIC, episodic cholestasis, DILI, ICP, HCC, elevated LFT, gallstones, pruritus	
*TJP2*	TJP2	PFIC, episodic cholestasis, ICP, elevated LFT, gallstones, HCC, pruritus	Intestinal symptoms (diarrhoea), pancreatitis, hearing loss
*NR1H4*	FXR	PFIC, ICP, gallstones	Vitamin K-independent coagulopathy
*USP53*	USP53	PFIC	Hearing loss
*LSR*	LSR	PFIC	Speech and developmental delays
*Myosin5B*	Myosin5B	PFIC, MVID	MVID, intestinal symptoms, neurodevelopmental delay and pyramidal syndrome
*SEMA7A*	Semaphorin 7A	PFIC, ICP, MASLD	
*VPS33B*		ARC syndrome, but also incomplete ARC with predominant cholestasis	Kidney and bone disease
*VIPAS39*	VIPAR	ARC syndrome	Kidney and bone disease
*ABCC12*	MRP9	Cholestasis, ductopenia	

ARC, arthrogryposis–renal dysfunction–cholestasis; DILI, drug-induced liver injury; GGT, gamma-glutamyltransferase; HCC, hepatocellular carcinoma; ICP, intrahepatic cholestasis of pregnancy; LFT, liver function tests; MASLD, metabolic dysfunction-associated steatotic liver disease; MVID, microvillus inclusion disease; PFIC, progressive familial intrahepatic cholestasis.

#### BSEP and FXR deficiencies

BA excretion across the canalicular membrane is critically determined by the amount of functionally active bile salt export pump (BSEP), encoded by *ABCB11,* in the canalicular membrane of hepatocytes.^11^^,^^12^ BSEP expression is regulated by the farnesoid X receptor (FXR, encoded by *NR1H4*).^13^^,^^14^ Biallelic mutations in *NR1H4* or in *ABCB11,* leading to a major reduction in BSEP function in the liver, result in early-onset and rapidly progressive PFIC phenotypes, characterised by normal levels of serum gamma-glutamyltransferase (GGT) despite high serum BA levels.^11^^,^[15], [16], [17], [18] In individuals of European descent, the two most common PFIC-associated BSEP variants are p.E297G and p.D482G, both of which retain some functional activity.^19^ The presence of at least one of these two variants, along with another missense variant on the other allele, can prolong native liver survival compared to persons carrying biallelic predicted protein truncating variants (PPTMs) and may underlie late-onset PFIC or even less severe phenotypes like recurrent cholestasis.^7^^,^^8^^,^^17^^,^^18^^,^^20^^,^^21^ Common variants in either *NR1H4* (-1G>T, rs56163822) or *ABCB11* (p.V444A, rs2287622) combined with deleterious heterozygous *ABCB11* variants may reduce BSEP protein expression and transport capacity to levels that enable adequate transport of BAs across the canalicular membrane under normal conditions.[22], [23], [24], [25] However, elevation of proinflammatory cytokines during bacterial or viral infections, elevation of progesterone and oestrogen levels during pregnancy, or intake of various drugs reduce already diminished BSEP levels further and thus below a threshold where BA secretion is impaired and cholestasis ensues.^2^^,^[26], [27], [28], [29], [30]

#### FIC1 deficiency

*ATP8B1* encodes familial intrahepatic cholestasis 1 (FIC1), a P-type ATPase, which flips phosphatidylserine from the outer into the inner leaflet of the plasma membrane in many cells, including in the canalicular membrane of hepatocytes.[31], [32], [33] FIC1 deficiency disturbs the lipid asymmetry within the canalicular membrane, affecting the transport activity of BSEP and rendering the membrane vulnerable to the detergent activity of BAs.^31^^,^^32^ It has also been suggested that FIC1 affects trafficking of hepatobiliary transport proteins, which may also contribute to cholestasis development.^2^^,^^33^^,^^34^ In contrast to BSEP deficiency, no correlation between mutation type (biallelic missense *vs.* biallelic PPTMs) and native liver survival has been observed so far.^17^^,^^35^

#### TJP2 deficiency

Tight junction protein 2 (TJP2, also known as zona occludens-2) isoforms are almost ubiquitously expressed in epithelial cells and form a link between the transmembrane components of the tight junction complexes, such as claudin-1 and claudin-2, and the actin cytoskeleton.^36^ A missense mutation (p.V48A) in TJP2 was first identified in Amish persons presenting with hypercholanaemia characterised by pruritus, elevated serum BAs and fat malabsorption but without progressive liver disease.^37^ *In vitro* studies suggested that this variant impairs the interaction of TJP2 with claudins.^37^ Subsequently, more damaging *TJP2* mutations were detected in patients with low-GGT PFIC.^38^ Mice with liver-specific deletion of *Tjp2* developed a mild cholestatic phenotype, which was aggravated by cholic acid feeding. Mechanistically, canaliculi were dilated and Bsep levels at the canalicular membrane were reduced, resulting in elevated serum BA levels.^39^ Induced pluripotent stem-cell-derived hepatocytes from a patient with TJP2 deficiency revealed distorted bile canaliculi and altered localisation of the apical and basolateral hepatobiliary transport proteins, including BSEP, resulting in impaired BA secretion across the apical membrane.^40^ Similar to patients with BSEP deficiency, patients carrying biallelic TJP2 protein truncating variants suffer from a rapidly progressive phenotype.^17^^,^^41^

#### USP53 deficiency

*USP53* encoding ubiquitin carboxyl-terminal hydrolase 53 is another gene associated with low-GGT PFIC in different cohorts.^42^^,^^43^ The function of USP53, which lacks deubiquitinase activity, and the pathophysiology underling USP53-associated cholestasis remain elusive.^43^^,^^44^ Usp53 was shown to interact with Tjp2 in a mouse model and Bsep expression levels were reduced in the livers of hepatocyte-specific *Usp53* knockout mice.^45^ Due to the more hydrophilic BA pool composition, mice deficient in PFIC-associated genes do not necessarily develop spontaneous (progressive) cholestasis and liver injury on a regular chow diet. However, BA feeding can trigger intrahepatic cholestasis and aggravate liver damage in some of these mouse models, including *Bsep*-, *Fic1*-and *Fxr*-deficient mice.^13^^,^^46^^,^^47^ Notably, hepatocyte-specific *Usp53*-deficient mice did not develop significant cholestasis or liver injury after cholic acid feeding in contrast to the *Tjp2*-deficient animals.^39^^,^^45^ Mice carrying a homozygous point mutation in *Usp53* were initially identified in a large-scale screen for novel recessive deafness traits.^48^ Importantly, TJP2 deficiency in humans has also been linked to hearing impairment.^49^^,^^50^

#### LSR deficiency

Pathogenic mutations in the gene encoding lipolysis-stimulated lipoprotein receptor (LSR) have recently been associated with PFIC.^42^^,^^51^ LSR is highly expressed in hepatocytes, binds apolipoprotein B/E-containing lipoproteins and plays a pivotal role in the formation of the tricellular tight junction complexes formed between three neighbouring cells.^52^^,^^53^ Mice with deletion of Lsr are not viable and show liver hypoplasia at the time of death.^53^ LSR deficiency in humans presents as low-GGT cholestasis and was associated with speech and developmental delay,^42^^,^^51^ which may be attributed to the high expression of LSR in the blood-brain barrier and central nervous system.

#### MYO5B deficiency

Mutations in the Myosin 5B (*MYO5B*) gene have been associated with microvillus inclusion disease, low-GGT intrahepatic cholestasis in the absence of intestinal symptoms, or a combination of both phenotypes.[54], [55], [56], [57], [58] While biallelic PPTM in *MYO5B* predispose to microvillus inclusion disease, mutations with residual function often manifest as intrahepatic cholestasis without overt intestinal disease.^59^^,^^60^ MYO5B plays a role in plasma membrane recycling, intracellular vesicular trafficking and cell polarisation in enterocytes and hepatocytes.^61^^,^^62^ In hepatocytes, MYO5B interacts with RAB11A to promote canalicular trafficking of hepatobiliary transporters, such as BSEP, MDR3 and MRP2. Immunohistochemistry revealed subcanalicular, and in some cases additionally, reduced staining for BSEP and RAB11 as a potential mechanism of primary cholestasis.^54^^,^^55^

#### SEMA7A deficiency

Semaphorin7A (*SEMA7A*) also known as CD108 is a membrane-bound protein expressed in different epithelial cells, including hepatocytes, that has been linked to immune cell activation, pulmonary fibrosis and cancer progression.^63^ A homozygous missense mutation was identified in a child with a PFIC phenotype and elevated serum BAs. Introducing the SEMA7A p.R148W mutation into a mouse model led to dose-dependent spontaneous elevation of serum BAs, aspartate aminotransferase (AST) and alanine aminotransferase (ALT) levels, as well as steatosis development.^63^^,^^64^ Mechanistically, increased phosphorylation of PKCδ/ε, as well as a reduction and intracellular retention of Bsep and Mrp2, was observed.^63^ Moreover, SEMA7A interacts and activates integrin α_5_β_1_,^65^^,^^66^ leading to insertion of Bsep into the canalicular membrane and promoting choleresis,^67^ which may be an alternative explanation for SEMA7A-associated cholestasis. Canalicular morphology and localisation of canalicular transporters is disturbed in mice with β1 integrin deficiency.^68^

#### ABCC12 deficiency

Another ABC transporter more recently linked to intrahepatic cholestasis is MRP9, encoded by the *ABCC12* gene.^69^ The substrates transported by MRP9 have not been elucidated. MRP9 is expressed in cholangiocytes and MRP9 deficiency is characterised by bile duct loss, but normal GGT cholestasis. The latter is surprising as deletion of *Abcc12* in zebrafish and mice resulted in increased cholangiocyte apoptosis, bile duct loss, elevated alkaline phosphatase (ALP) and liver fibrosis.^69^

#### Cholestasis-associated genes affecting intracellular trafficking

*VPS33B, VIPAS39* (encoding VIPAR), *AP1S1* and *SCYL1*, similarly to *MYO5B*, are associated with intracellular trafficking and recycling of endosomes.^70^ Mutations in these genes have been linked to low-GGT primary cholestasis and additionally predispose individuals to rare multisystem cholestasis syndromes, such as arthrogryposis–renal dysfunction–cholestasis (*VPS33B, VIPAS39*),^71^^,^^72^ MEDNIK (mental retardation, enteropathy, deafness, peripheral neuropathy, ichthyosis, keratoderma; *AP1S1*)^73^ and CALFAN (with low-GGT cholestasis, acute liver failure, and neurodegeneration; *SCYL1*).^74^ VPS33B deficiency leads to intracellular retention of BSEP and thus primary cholestasis.^75^

### Genes associated with cholangiopathies and elevated GGT levels

Mutations in genes associated with cholangiopathies include *ABCB4*, *KIF12*, *ZFYVE19*, *CLDN1*, *DCDC2*, *PLEC1*, *UNC5A*, *JAG1* and *NOTCH2*.^9^ These are characterised by biliary damage and elevated GGT levels (Table 2).

**Table 2:** Genes associated with cholangiopathies usually characterised by elevated GGT and ALP (modified from^9^).

Gene	Protein	Clinical manifestations	Extrahepatic manifestations
*ABCB4*	MDR3	PFIC, sclerosing cholangitis, biliary fibrosis, LPAC, gallstones, ICP, CCA, elevated LFTs	
*KIF12*	KIF12	PFIC, elevated LFTs	Normal renal function
*ZFYVE19*	ZFYVE19	Neonatal sclerosing cholangitis	
*CLDN1*	Claudin 1	Neonatal ichthyosis–sclerosing cholangitis (NISCH) syndrome, sclerosing cholangitis	Hypotrichosis, alopecia, ichthyosis
*DCDC2*	Doublecortin domain containing protein 2	Neonatal sclerosing cholangitis	Renal disease, nephronophthisis
*PLEC*	Plectin	PFIC	Plectinopathies, including epidermolysis, muscular dystrophy
*PSKH1*	Protein serine kinase H1	Cholangiopathy, biliary fibrosis	Glomerulopathy, developmental delay
*JAG1*	Jagged 1	Alagille syndrome	Features of Alagille
*NOTCH2*	NOTCH2	Alagille syndrome	Features of Alagille

ALP, alkaline phosphatase; CCA, cholangiocarcinoma; GGT, gamma-glutamyltransferase; LFTs, liver function tests; LPAC, low phospholipid-associated cholelithiasis; PFIC, progressive familial intrahepatic cholestasis.

#### MDR3 deficiency

*ABCB4* encodes the multidrug-resistance protein 3 (MDR3), which is a phospholipid floppase essential for the extraction of phosphatidylcholine into bile.^76^^,^^77^ Together with BAs, phosphatidylcholine forms mixed micelles increasing the solubility of cholesterol in bile and protecting the biliary epithelium from the detergent activity of bile acids.^78^^,^^79^ Impaired MDR3 function leads to free BAs in bile, and subsequent cholangiocyte damage, resulting in progressive biliary fibrosis and/or PFIC with elevated GGT levels.^80^^,^^81^ Furthermore, the reduced capacity to form mixed micelles increases the risk of gallstone disease and low phospholipid-associated cholelithiasis syndrome.[81], [82], [83], [84], [85], [86] *Mdr2*-deficient mice spontaneously develop gallstones, a progressive liver disease characterised by periductal fibrosis and biliary strictures, and hepatobiliary malignancy.^79^ While patients carrying biallelic *ABCB4* PPTMs usually present early in life with PFIC, less severe mutations manifest in adults as intrahepatic cholestasis or pregnancy (ICP), elevated GGT, gallstone disease, biliary fibrosis and hepatobiliary malignancy.^7^^,^^80^^,^^81^^,^[86], [87], [88], [89], [90] Compared to patients with biallelic PPTMs, the presence of at least one *ABCB4* missense mutation and biliary phospholipid levels greater than 6.9% of total biliary lipids was predictive of a response to ursodeoxycholic acid (UDCA) and prolonged native liver survival.^90^

#### KIF12 deficiency

Kinesin related protein 12 (KIF12) is putatively a microtubule-associated motor protein predicted to play a role in intracellular trafficking and cytoskeleton organisation.^91^^,^^92^ Biallelic mutations in *KIF12* were identified in infants with high-GGT PFIC (cholangiopathy).^42^ In comparison to serum BAs, GGT levels were severely elevated.^42^^,^^93^^,^^94^ Immunohistochemistry of livers from patients with KIF12 deficiency revealed a reduced apical localisation of BSEP and MRP2 similar to MYO5B deficiency. However, the mechanisms leading to the marked GGT elevation and a more pronounced cholangiopathic presentation are unclear.

#### ZFYVE19 deficiency

Mutations in the zinc finger FYVE-type containing 19 (*ZFYVE19*) gene have been described in patients with high-GGT PFIC characterised by biliary tract abnormalities and congenital fibrosing cholangiopathy.^95^ *Zfyve19*-deficient mice do not develop liver disease on regular chow diet; however, after exposure to α-naphthyl isothiocyanate, markers of liver injury were elevated in serum (ALT, ALP and BAs) and abnormal cholangiocyte polarity and portal fibrosis were observed on histology.^96^ Impaired centrosome splitting and cell division was detected in isolated cells, which was most likely responsible for the increased cholangiocyte apoptosis.^96^ Similarly, disruption of primary cilia structure and altered centriole number and localisation have been described in patient-derived fibroblasts.^97^

#### Further genes associated with cholangiopathy development

Ciliopathies presenting as sclerosing cholangitis together with different extrahepatic manifestations have been attributed to mutations in the genes encoding claudin 1 (*CLDN1*) and doublecortin domain containing protein 2 (*DCDC2*).[98], [99], [100] Moreover, mutations in the *PSKH1* and *PLEC* genes may predispose to an early-onset cholangiopathy and cholestasis^101^^,^^102^ and mutations in *JAG1* and *NOTCH2* underlie Alagille syndrome.^103^^,^^104^

## Clinical presentations associated with genetic cholestasis in adults

The clinical presentation of adults carrying pathogenic mutations in the genes mentioned above is highly variable. In fact, even within one family different phenotypes have been reported in individuals carrying the same mutation.^7^^,^^8^^,^^105^^,^^106^ Patients with biallelic PPTMs usually present early in life with PFIC. Biallelic variants with residual function and heterozygous variants tend to manifest as milder phenotypes. Patients may present with elevated liver enzymes, pruritus, episodic cholestasis, ICP, drug-induced liver injury, gallstones/low phospholipid-associated cholelithiasis syndrome or more severe late-onset PFIC, progressive liver disease and hepatobiliary malignancies, or combinations thereof.^7^^,^^8^^,^^105^^,^^107^^,^^108^ For several genetic cholangiopathies, but especially for MDR3 deficiency, clinical presentation and features may resemble primary sclerosing cholangitis (PSC) or Caroli disease/syndrome, with dilatation of intrahepatic ducts on both magnetic resonance cholangiopancreatography and histology.^87^^,^[108], [109], [110] Pruritus and fatigue are common symptoms not only of primary biliary cholangitis (PBC) but also of hereditary cholestasis; thus, some of these patients may be mislabelled as having anti-mitochondrial antibody/anti-nuclear antibody-negative (seronegative) PBC.^6^^,^^111^

## Diagnosis of genetic cholestasis in adults

**Fig. 3:**
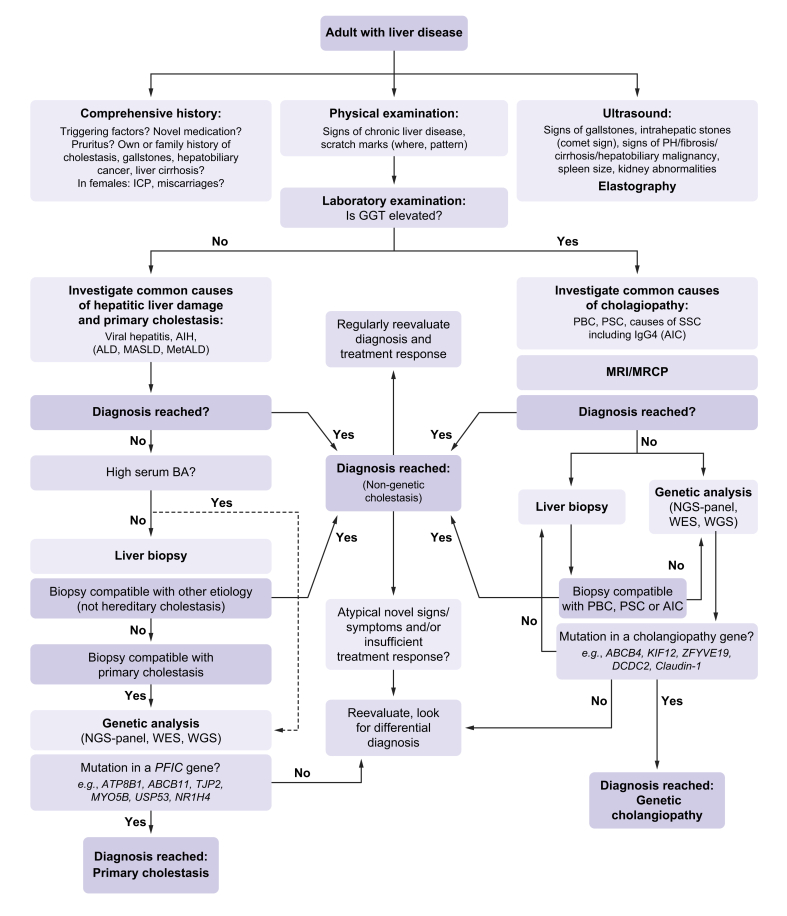
Approach to adults with liver disease. AIC, autoimmune cholestasis; AIH, autoimmune hepatitis; ALD, alcohol-related liver disease; BA, bile acid; ICP, intrahepatic cholestasis of pregnancy; MASLD, metabolic dysfunction-associated steatotic liver disease; MetALD, metabolic- and alcohol-associated steatotic liver disease; NGS, next-generation sequencing; PBC, primary biliary cholangitis; PSC, primary sclerosing cholangitis; SSC, secondary sclerosing cholangitis; MRCP, magnetic resonance cholangiopancreatography; WES, whole-exome sequencing; WGS, whole-genome sequencing.

The large overlap in clinical presentation, imaging and sometimes even histological findings of more common liver diseases with hereditary primary cholestasis and cholangiopathies warrants a stepwise approach to diagnosis (Fig. 3). As a first step, low-GGT primary cholestasis, which is characterised by elevated ALT, AST, BAs, and often bilirubin, should be distinguished from high-GGT cholangiopathy. In both cases diagnostic workup for more common liver diseases should precede genetic analysis. Most patients will receive a diagnosis during this workup. Those patients who remain undiagnosed should undergo genetic testing and/or liver biopsy.

Reduced sequencing costs and the increased availability of clinical genomic testing, both commercially and through academic-affiliated laboratories, have made genomic analysis more accessible in clinical practice. Nevertheless, there remains an unmet need for increased education and awareness of adult-presenting genetic cholestatic liver diseases, particularly in non-tertiary academic medical centres. When a hepatologist suspects a genetic liver disease, they can opt to order a gene panel test, WES or even whole-genome sequencing.^112^^,^^113^ Clinical geneticists search for rare genetic variants (typically with a frequency of less than 1% in the general population for recessive inheritance) in genes known to cause cholestasis and predict their impact on protein function. Given the ongoing identification of new liver disease-associated genes,^114^ gene panels require regular updating to include newly discovered genes, whereas WES captures and sequences nearly all coding regions of approximately 20,000 human protein coding genes. WES workup usually includes a first analysis with a predefined virtual gene panel (similar to a next-generation sequencing panel) and, if the diagnosis remains unclear, can be extended to a more in-depth analysis of all coding regions. In addition, negative genetic testing does not fully exclude an underlying genetic condition. Re-analysis of WES data at later time points can cost-effectively increase the diagnostic yield (∼10-30% additional variants in disease-associated or candidate genes),^115^^,^^116^ primarily through the identification of newly discovered genetic disorders. Hence, WES re-analysis is recommended for patients with unexplained cholestasis every 1 to 2 years, but at least every 3 years.^112^ Gene panels and WES primarily identify variants in coding regions of the human genome. Studies are required to investigate the burden and spectrum of single nucleotide variants, indels and structural variations in non-coding regulatory regions that are not captured by gene panels or WES. Moreover, emerging evidence implicates somatic mutations in rare genetic liver diseases, such as in alpha-1-antitrypsin deficiency,^117^^,^^118^ though their role in cholestatic disorders remains to be explored.

**Fig. 4:**
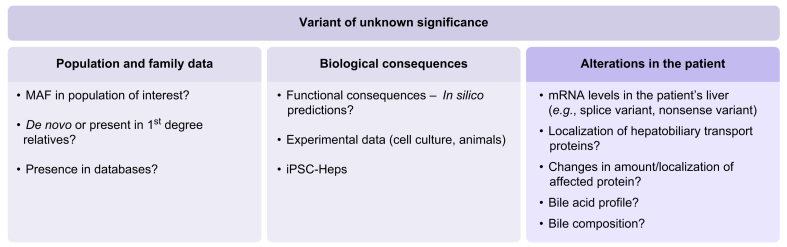
Factors to consider when evaluating the potential clinical relevance of a variant of unknown significance. While the left boxes (light grey) are evaluated by the geneticist and underlie the classification as variants of unknown significance, the right column (light blue) contains factors that can be assessed by the clinician for further evaluation of a variant of unknown significance. MAF, minor allele frequency.

A challenge with gene panel tests and/or WES in diagnosing and managing adult liver disease is the identification of genetic variants of unknown significance (VUS). This classification is based on criteria put forward by the ACMG (American College of Medical Genetics and Genomics). These criteria include information on the inheritance of the variant (monoallelic or bi-allelic), allelic frequency in the general population (allelic frequency in affected and healthy individuals), *in silico* computational predictive data and available (experimental) functional data for likely or definitive pathogenicity. As more data become available, VUS can be reclassified as either pathogenic or benign. In some cases, additional investigations of clinically accessible tissue or samples may allow for reclassification of VUS. Examples are testing of hepatic mRNA levels in a suspected splice variant or determining biliary BA or phospholipid levels in an *ABCB11* or *ABCB4* VUS. Patient-derived induced pluripotent stem-cell-derived hepatocytes may also enable further functional characterisation of a VUS; however, they are only available for a limited number of cases^40^ (Fig. 4). The key question in interpreting genetic test results is whether the identified variants can fully or at least partly explain the patient’s clinical phenotype. To address this diagnostic challenge, a few centres have established multidisciplinary genome rounds, which integrate clinical and genetic information to achieve a definitive diagnosis. This approach relies on the expertise of adult and paediatric hepatologists, liver pathologists and clinical geneticists and has direct implications for optimising patient management, refining prognostic assessment and guiding family counselling.^119^

A liver biopsy can support a role of the genetic abnormality in the clinical phenotype if lesions are compatible with the known histological effect of the protein deficiency. Histology results can provide insightful information to better understand the consequences of the genetic defects in the liver tissue in this heterogeneous group of patients. Although there is individual variability in the phenotypic expression at the tissue level among patients with the same variants, liver biopsy remains an effective tool to provide integrative information on clinical, genetic, and histological findings.^119^

## Pathology

None of the lesions described in genetic cholestasis are specific to these diseases; therefore, the combination of clinical/biochemical/serological/radiological context is essential for correct diagnosis.^8^ When a genetic abnormality is known, the role of liver biopsy is to support or refute the potentially harmful effect of the gene variant(s) based on currently available information. When the biopsy is performed before available genetic results there are some features that should raise concern and prompt genetic cholestasis testing. The EASL CPG for genetic cholestasis recommends that a liver biopsy should be considered when careful non-invasive evaluation remains inconclusive.^112^

In primary cholestasis, the injury will primarily affect hepatocytes, with abnormal/excessive accumulation of toxic bile components within the cell, which may be represented histologically by visualisation of bile pigment (so-called bilirubinostasis) in the hepatocyte cytoplasm and more typically within dilated canaliculi. This can be associated with cytopathic damage of hepatocytes, characterised by loss of the polygonal shape, swelling and clear appearance of the cytoplasm, changes in cell arrangement with cholestatic rosettes, and hepatocellular multinucleation. If these changes are not associated with significant portal or lobular inflammation the diagnosis of bland cholestasis is made. This histological pattern is typical for ICP and recurrent intrahepatic cholestasis. Bland cholestasis can be seen in other clinical settings such as septic conditions, ischaemia, and some paraneoplastic manifestations (*e.g*. Hodgkin’s lymphoma), although the latter cannot exclude an underlying genetic abnormality.

Cholestatic hepatitis is defined by lobular bile stasis in association with a lobular hepatitis component. In this situation portal inflammation and bile duct injury may also be present. This pattern of injury is typical for drug-induced liver injury. It can be present in other aetiologies of hepatitis, such as viral or autoimmune hepatitis, but in these contexts the necroinflammatory injury component dominates the histological picture.

Cholangiopathy in adult-onset genetic cholestasis may show overlapping features with PSC, or early-stage PBC when granulomas are absent. Normal biliary tree imaging and absence of typical serologic autoimmune markers would support a potential genetic cholestasis. Cases previously reported as small duct PSC or atypical forms of PBC (anti-mitochondrial antibody-negative, ductopenic PBC in young patients) may in fact represent phenotypic forms of PFIC. MDR3 deficiency is the most common genetic condition in adult patients with biopsy-proven cholangiopathy, while less commonly affected genes, such as *KIF12*, should also be considered. Liver biopsy in MDR3 deficiency (usually in a patient with dominant elevation of ALP and GGT with/without hypertransaminasemia) may reveal focal portal involvement and variable disease stages, ranging from subtle bile duct damage in a minority of portal tracts to established chronic biliary disease with portal fibrosis or cirrhosis. In early stages, the histopathologist should carefully examine every portal area for bile duct abnormalities, given the mild and focal bile duct changes (*e.g.* cholangiocyte disarray or focal cell loss, thickening of the bile duct basement membrane, periductal fibrosis, or focal bile duct loss) can easily be overlooked.^87^^,^^107^ Features of chronic cholestasis, which can also be very focal, are extremely helpful in supporting the diagnosis of early or established chronic biliary disease. In addition to cholate stasis in periportal hepatocytes, the use of special stains and immunohistochemistry are invaluable histological tools to demonstrate periportal copper or copper-associated protein deposition or periportal hepatocellular cytokeratin 7 expression.^120^

Any biopsy with “biliary features”, either bile duct injury with or without bile duct loss, biliary proliferation, and/or identification of periportal copper-associated protein or aberrant hepatocellular expression of cytokeratin 7, independent of the degree of fibrosis observed, should prompt consideration of the possibility of an underlying genetic condition in patients with normal magnetic resonance cholangiopancreatography and absence of typical autoimmune serology. In the exceptional situation where cholesterol clefts or intrahepatic lithiasis are identified within the biopsied bile ducts, the most likely diagnosis is MDR3 deficiency.^121^

After a genetic or non-genetic diagnosis is reached, patients should be monitored for new symptoms and signs, as well as treatment response. If novel findings occur or the response to treatment is incomplete, consider potential additional or alternative causes including re-entering the patient into the diagnostic workup algorithm. Sometimes a re-analysis of the histopathology or genetic findings can reveal novel aspects underappreciated on the initial evaluation.^4^ Furthermore, the adult-onset presentation of known paediatric cholestatic liver diseases has only recently been recognised; prospective data will provide insight into their natural history.

## Treatment of genetic cholestasis in adults

There have been no randomised controlled trials in adult-onset genetic cholestasis. The strongest evidence supporting treatment choices comes from paediatric trials, retrospective cohort studies and empirical treatment. Management of genetic cholestasis should best be thought of at several levels. The ideal treatment should target the underlying pathology, and in this respect a clear understanding of the pathophysiology in each patient is essential. If targeted treatment is not possible, treatment for cholestasis may be required, and finally treatment of the consequences of cholestasis may be necessary; this should include symptom control and nutritional support.

Mutation- or gene-specific treatments are still pre-clinical for these diseases. In most cases it is likely that severe early-onset phenotypes will be treated in initial trials. However, inclusion of adult cohorts will be essential if therapy is to be made available to adult patients in the future. Targeted therapy, for now, consists of addressing the major components of the diseases, as discussed above. The retention of components of bile in hepatocytes, along with its consequences, is a feature common to nearly all these conditions. However, the potentially toxic milieu of bile may be modified in the case of the cholangiopathies.

UDCA has been widely used in cholestatic diseases, genetic or otherwise. It is itself a BA and might even be harmful in severe cholestasis. However, there are suggestions that it can increase BSEP expression, which would be beneficial in many conditions.^122^^,^^123^ The evidence of clinical benefit is missing. The only good evidence for the use of UDCA is in non-genetic cholangiopathies, such as PBC, and in MDR3 deficiency.^90^^,^[124], [125], [126] In the latter, BA toxicity in bile is at the heart of the disease, and reducing the hydrophobicity of the BA pool is entirely logical. Retrospective data show that biochemical improvement, after the introduction of UDCA in MDR3 deficiency, improves outcomes.^90^^,^^126^ The use of other hydrophilic BAs, such as norucholic acid, is logical, but unproven.

Depletion of BA pool size, by interruption of the enterohepatic circulation of BAs or through inhibition of BA synthesis, is a logical intervention in most forms of cholestasis. One possible benefit might be a reduction of BA retention in the liver and a consequent reduction in liver damage and symptoms such as pruritus. The second benefit might be enrichment of the BA pool and enhancement of the effect of hydrophilic BA treatment. The latter is not yet proven. Depletion of the BA pool has been undertaken mechanically, via partial external biliary diversion or nasobiliary drainage.^17^^,^^112^^,^^127^^,^^128^ Historically the only non-invasive option has been BA-binding resins. These have limited value, and suffer from reducing the availability of BAs in the small bowel, and from binding lipophilic dietary components, notably fat-soluble vitamins.^112^ Recent paediatric studies have shown considerable efficacy from the use of inhibitors of ASBT (apical sodium dependent bile salt transporter), also known as IBAT (ileal bile acid transporter). Randomised controlled trials of both odevixibat and maralixibat in various types of PFIC have shown efficacy and tolerability.[129], [130], [131], [132] These drugs are now licensed for the treatment of PFIC in many countries. The adult diseases discussed in this article are milder versions of the diseases studied in the pivotal trials. It is logical to think that these drugs will work in milder phenotypes.

FXR agonists have not been systematically explored in genetic cholestasis to date. They may aid BA depletion, potentially in combination with other treatments. However, in cholestasis, there is already a high level of hepatocyte FXR activation from the accumulated BAs.

Bezafibrate, which is approved for the treatment of dyslipidaemia, has been successfully used as second-line therapy in patients with PBC and an incomplete response to UDCA. Besides reduction in ALP levels, bezafibrate significantly reduced pruritus in patients with PBC and PSC.^133^ ^134^ Treatment of dyslipidaemia with bezafibrate in children with FIC1 deficiency alleviated pruritus in all three patients.^135^ A reduction in pruritus in response to bezafibrate has also been observed in individual patients with BSEP deficiency (VK personal communication), but clinical trial data are lacking.

Prior to the availability of targeted drugs, the mainstays of management for these conditions were focused on symptoms and the prevention of complications. Treatment of pruritus, as opposed to treatment of the underlying disease, remains unsatisfactory. In addition to UDCA, rifampicin, ondansetron, naltrexone and selective serotonin reuptake inhibitors have all been used. None have been subject to rigorous trials. Monitoring for fat-soluble vitamin deficiency, and supplementation where necessary, is essential. Nutritional support might also be needed.

Monitoring for complications of chronic liver disease is essential in these conditions. Cholangiopathies, MDR3 deficiency in particular, have been strongly associated with the development of both hepatocellular carcinoma and cholangiocarcinoma. Indeed, hepatobiliary malignancy is the initial presentation in some cases.

## Conclusions

Genetic defects are increasingly recognised as causative or relevant contributing factors in adult cholestatic liver disease. The broad spectrum of clinical presentations poses a major challenge in reaching a diagnosis; therefore, a stepwise approach is suggested. Management of genetic cholestasis should target the underlying pathology whenever possible, should alleviate the consequences of cholestasis and should include symptom control.

Lastly, given the insidious nature of some adult-onset genetic cholestatic liver diseases and the presence of disease in individuals who are only heterozygous for disease-causing mutations, we suggest screening heterozygous parents for liver disease at the time of their child’s diagnosis.

## Abbreviations

ABCB4/11, ABC transporter B4/11; ALP, alkaline phosphatase; ALT, alanine aminotransferase; AST, aspartate aminotransferase; ATP8B1, ATPase phospholipid transporting 8B1; BA, bile acid; BSEP, bile salt export pump; FIC1, familial intrahepatic cholestasis 1; FXR, farnesoid X receptor; GGT, gamma-glutamyltransferase; ICP, intrahepatic cholestasis of pregnancy; KIF12, kinesin related protein 12; LPAC, low phospholipid-associated cholelithiasis; LSR, lipolysis-stimulated lipoprotein receptor; MYO5B, myosin 5B; NR1H4, nuclear receptor 1H4; PBC, primary biliary cholangitis; PFIC, progressive familial intrahepatic cholestasis; PPTM, predicted protein truncating mutation; PSC, primary sclerosing cholangitis; SEMA7A, semaphorin 7A; TJP2, tight junction protein 2; UDCA, ursodeoxycholic acid; USP53, ubiquitin carboxyl-terminal hydrolase 53; VUS, variant of unknown significance; WES, whole-exome sequencing; ZFYVE19, zinc finger FYVE-type containing 19.

## Financial support

SV is supported by NIDDK, R01 DK131033, VK is supported by BMBF through HiChol (01GM1904A and 01GM2204A).

## Authors’ contributions

VK designed the manuscript and created the figures. RT, RM, SV and VK created the diagnostic algorithm. VK, RT, RM, SV wrote the manuscript and all approved the final version.

## Conflict of interest

No author received funding for this publication. RJT has consulted for Mirum, Ipsen, Alnylam, Spruce Bio, Glycomine, Rectify, and Generation Bio. He has received speaker’s honoraria from Mirum and Ipsen. He has stock/options in Rectify, Generation Bio and Integra. VK received honoraria for speaker’s corner from Falk, Mirum, Ipsen, GSK, Gilead and participated in advisory boards by Mirum, Ipsen, Falk and GSK. SV served as consultant for Mirum and Ipsen and received research grant support from Moderna.

Please refer to the accompanying ICMJE disclosure forms for further details.
